# 4-Chloro-6-meth­oxy­pyrimidin-2-amine–succinic acid (2/1)

**DOI:** 10.1107/S1600536812046156

**Published:** 2012-11-14

**Authors:** Kaliyaperumal Thanigaimani, Nuridayanti Che Khalib, Suhana Arshad, Ibrahim Abdul Razak

**Affiliations:** a School of Physics, Universiti Sains Malaysia, 11800 USM, Penang, Malaysia

## Abstract

The asymmetric unit of the title compound, 2C_5_H_6_ClN_3_O·C_4_H_6_O_4_, consists of one 4-chloro-6-meth­oxy­pyrimidin-2-amine mol­ecule and one half-mol­ecule of succinic acid which lies about an inversion centre. In the crystal, the acid and base mol­ecules are linked through N—H⋯O and O—H⋯N hydrogen bonds, forming a tape along [1-10] in which *R*_2_^2^(8) and *R*_4_^2^(8) hydrogen-bond motifs are observed. The tapes are further inter­linked through a pair of C—H⋯O hydrogen bonds into a sheet parallel to (11-2).

## Related literature

For applications of pyrimidine derivatives, see: Condon *et al.* (1993 ▶); Maeno *et al.* (1990 ▶); Gilchrist (1997 ▶). For applications of succinic acid, see: Zeikus *et al.* (1999 ▶); Song & Lee (2006 ▶). For hydrogen-bond motifs, see: Bernstein *et al.* (1995 ▶). For bond-length data, see: Allen *et al.* (1987 ▶). For stability of the temperature controller used for the data collection, see: Cosier & Glazer (1986 ▶).
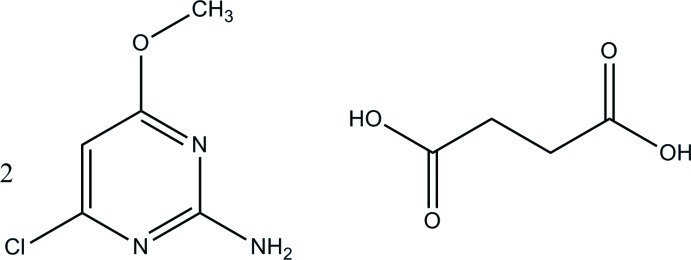


## Experimental

### 

#### Crystal data

2C_5_H_6_ClN_3_O·C_4_H_6_O_4_
*M* *_r_* = 437.24
Triclinic, 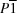

*a* = 5.0094 (2) Å
*b* = 8.5459 (4) Å
*c* = 10.8736 (5) Å
α = 82.337 (1)°
β = 88.952 (1)°
γ = 86.904 (1)°
*V* = 460.64 (4) Å^3^
*Z* = 1
Mo *K*α radiation
μ = 0.40 mm^−1^
*T* = 100 K
0.60 × 0.22 × 0.14 mm

#### Data collection

Bruker SMART APEXII DUO CCD area-detector diffractometer
Absorption correction: multi-scan (*SADABS*; Bruker, 2009 ▶) *T*_min_ = 0.796, *T*_max_ = 0.945
7766 measured reflections
1875 independent reflections
1808 reflections with *I* > 2σ(*I*)
*R*_int_ = 0.016

#### Refinement

*R*[*F* ^2^ > 2σ(*F* ^2^)] = 0.024
*wR*(*F* ^2^) = 0.069
*S* = 1.09
1875 reflections
140 parameters
H atoms treated by a mixture of independent and constrained refinement
Δρ_max_ = 0.33 e Å^−3^
Δρ_min_ = −0.26 e Å^−3^

### 

Data collection: *APEX2* (Bruker, 2009 ▶); cell refinement: *SAINT* (Bruker, 2009 ▶); data reduction: *SAINT*; program(s) used to solve structure: *SHELXTL* (Sheldrick, 2008 ▶); program(s) used to refine structure: *SHELXTL*; molecular graphics: *SHELXTL*; software used to prepare material for publication: *SHELXTL* and *PLATON* (Spek, 2009 ▶).

## Supplementary Material

Crystal structure: contains datablock(s) global, I. DOI: 10.1107/S1600536812046156/is5213sup1.cif

Structure factors: contains datablock(s) I. DOI: 10.1107/S1600536812046156/is5213Isup2.hkl

Supplementary material file. DOI: 10.1107/S1600536812046156/is5213Isup3.cml

Additional supplementary materials:  crystallographic information; 3D view; checkCIF report

## Figures and Tables

**Table 1:** Hydrogen-bond geometry (Å, °)

*D*—H⋯*A*	*D*—H	H⋯*A*	*D*⋯*A*	*D*—H⋯*A*
N3—H1*N*3⋯O3	0.847 (17)	2.223 (17)	3.0055 (13)	153.7 (14)
N3—H2*N*3⋯O3^i^	0.844 (16)	2.095 (16)	2.9369 (13)	175.4 (15)
O2—H1*O*2⋯N2^i^	0.806 (16)	1.923 (16)	2.7266 (13)	174.6 (18)
C3—H3*A*⋯O1^ii^	0.95	2.45	3.3911 (14)	172

Symmetry codes: (i) 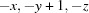; (ii) 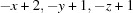.

**Fig. 1.**
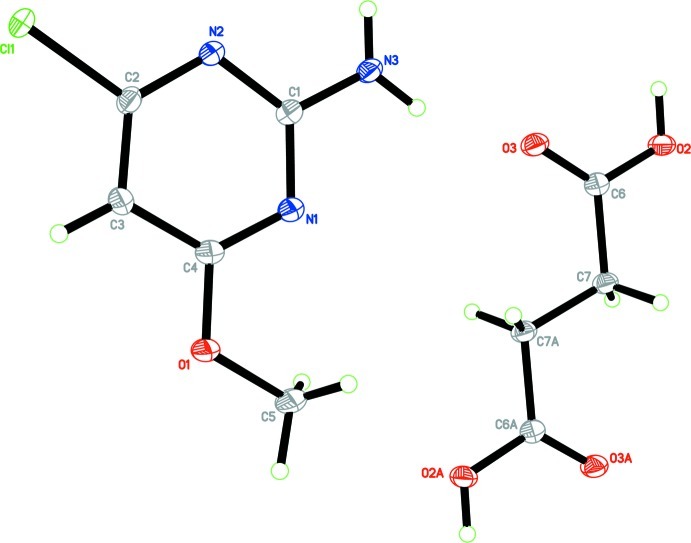
The molecular structure of the title compound with atom labels with 50% probability displacement ellipsoids.

**Fig. 2.**
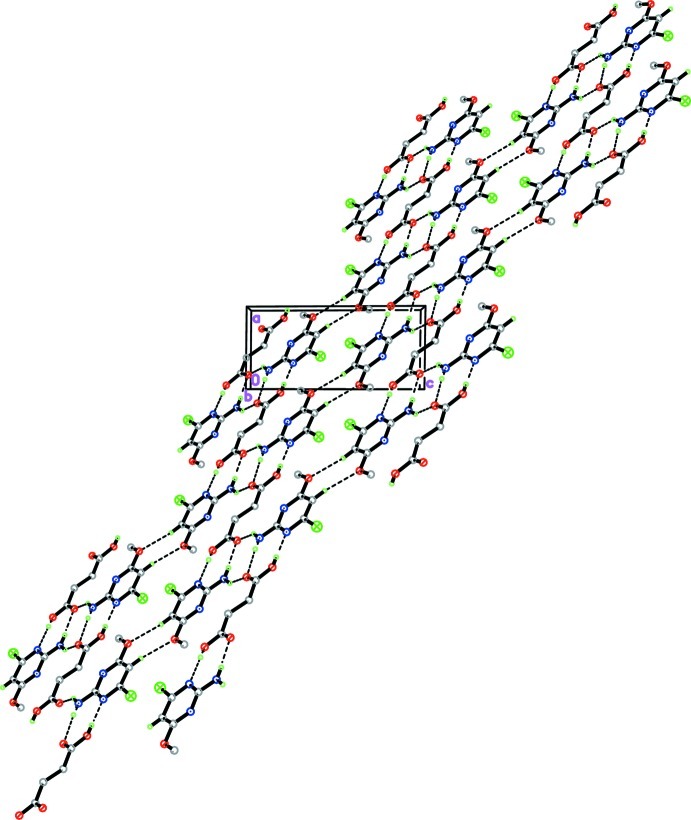
The crystal packing of the title compound. The H atoms not involved in the intermolecular interactions (dashed lines) have been omitted for clarity.

## supplementary crystallographic information

### Comment

Pyrimidine derivatives are very important molecules in biology and have many application in the areas of pesticide and pharmaceutical agents (Condon *et al.*, 1993). For example, imazosulfuron, ethirmol and mepanipyrim have been commercialized as agrochemicals (Maeno *et al.*, 1990). Pyrimidine derivatives have also been developed as antiviral agents, such as AZT, which is the most widely-used anti-AIDS drug (Gilchrist, 1997). The dicarboxylic acid, succinic acid, is a precursor for many chemicals of industrial importance (Zeikus *et al.*, 1999; Song & Lee, 2006). In order to study some interesting hydrogen bonding interactions, the synthesis and structure of the title compound, (I), is presented here.

The asymmetric unit of the title compound consists of a 4-chloro-6-methoxypyrimidin-2-amine molecule and a half of the succinic acid molecule (Fig. 1). The acid molecule is lying about an inversion centre. The 4-chloro-6-methoxypyrimidin-2-amine molecule is approximately planar, with a maximum deviation of 0.037 (1) Å for atom O1. The bond lengths (Allen *et al.*, 1987) and angle are normal.

In the crystal packing, the 4-chloro-6-methoxypyrimidin-2-amine molecules interact with the carboxylic group of the respective succinic acid molecules through N3—H2N3···O3^i^ and O2—H1O2···N2^i^ hydrogen bonds (symmetry code in Table 1), forming a hydrogen-bonded ring motif *R*_2_^2^(8) (Bernstein *et al.*, 1995). These motifs are centrosymmetrically paired *via* N3—H2N3···O3 hydrogen bonds, forming a complementary DADA array. These arrays are further interlinked with a neighboring array through a couple of C3—H3A···O1^ii^ hydrogen bonds (symmetry code in Table 1) combine together to form a large ring motif, with graph-set notation *R*_6_^6^(34). These ring motifs extend to give a sheet parallel to (112) plane as shown in Fig. 2.

### Experimental

Hot methanol solutions (20 ml) of 4-chloro-6-methoxypyrimidin-2-amine (36 mg, Aldrich) and succinic acid (29 mg, Merck) were mixed and warmed over a heating magnetic stirrer hotplate for a few minutes. The resulting solution was allowed to cool slowly at room temperature and crystals of the title compound (I) appeared after a few days.

### Refinement

O- and N-bound H atoms were located in a difference Fourier map and refined freely [refined distances: N—H = 0.846 (17) and 0.842 (18) Å, O—H = 0.804 (19) Å]. The remaining hydrogen atoms were positioned geometrically (C—H= 0.95–0.99 Å) and were refined using a riding model, with *U*_iso_(H) = 1.2*U*_eq_(C) or 1.5*U*_eq_(methyl C). A rotating group model was used for the methyl group. Three outliers were omitted (-4 5 3, -1 2 1 and 1 0 1) in the final refinement.

### Crystal data

**Table:**

2C_5_H_6_ClN_3_O·C_4_H_6_O_4_	*Z* = 1
*M**_r_* = 437.24	*F*(000) = 226
Triclinic, *P*1	*D*_x_ = 1.576 Mg m^−^^3^
Hall symbol: -P 1	Mo *K*α radiation, λ = 0.71073 Å
*a* = 5.0094 (2) Å	Cell parameters from 8335 reflections
*b* = 8.5459 (4) Å	θ = 3.3–32.6°
*c* = 10.8736 (5) Å	µ = 0.40 mm^−^^1^
α = 82.337 (1)°	*T* = 100 K
β = 88.952 (1)°	Block, colourless
γ = 86.904 (1)°	0.60 × 0.22 × 0.14 mm
*V* = 460.64 (4) Å^3^	

### Data collection

**Table:**

Bruker SMART APEXII DUO CCD area-detector diffractometer	1875 independent reflections
Radiation source: fine-focus sealed tube	1808 reflections with *I* > 2σ(*I*)
Graphite monochromator	*R*_int_ = 0.016
φ and ω scans	θ_max_ = 26.5°, θ_min_ = 1.9°
Absorption correction: multi-scan (*SADABS*; Bruker, 2009)	*h* = −6→6
*T*_min_ = 0.796, *T*_max_ = 0.945	*k* = −10→10
7766 measured reflections	*l* = −13→13

### Refinement

**Table:**

Refinement on *F*^2^	Primary atom site location: structure-invariant direct methods
Least-squares matrix: full	Secondary atom site location: difference Fourier map
*R*[*F*^2^ > 2σ(*F*^2^)] = 0.024	Hydrogen site location: inferred from neighbouring sites
*wR*(*F*^2^) = 0.069	H atoms treated by a mixture of independent and constrained refinement
*S* = 1.09	*w* = 1/[σ^2^(*F*_o_^2^) + (0.0384*P*)^2^ + 0.1625*P*] where *P* = (*F*_o_^2^ + 2*F*_c_^2^)/3
1875 reflections	(Δ/σ)_max_ < 0.001
140 parameters	Δρ_max_ = 0.33 e Å^−^^3^
0 restraints	Δρ_min_ = −0.26 e Å^−^^3^

### Special details

**Table:**

Experimental. The crystal was placed in the cold stream of an Oxford Cryosystems Cobra open-flow nitrogen cryostat (Cosier & Glazer, 1986) operating at 100.0 (1) K.
Geometry. All e.s.d.'s (except the e.s.d. in the dihedral angle between two l.s. planes) are estimated using the full covariance matrix. The cell e.s.d.'s are taken into account individually in the estimation of e.s.d.'s in distances, angles and torsion angles; correlations between e.s.d.'s in cell parameters are only used when they are defined by crystal symmetry. An approximate (isotropic) treatment of cell e.s.d.'s is used for estimating e.s.d.'s involving l.s. planes.
Refinement. Refinement of *F*^2^ against ALL reflections. The weighted *R*-factor *wR* and goodness of fit *S* are based on *F*^2^, conventional *R*-factors *R* are based on *F*, with *F* set to zero for negative *F*^2^. The threshold expression of *F*^2^ > σ(*F*^2^) is used only for calculating *R*-factors(gt) *etc*. and is not relevant to the choice of reflections for refinement. *R*-factors based on *F*^2^ are statistically about twice as large as those based on *F*, and *R*- factors based on ALL data will be even larger.

### Fractional atomic coordinates and isotropic or equivalent isotropic displacement parameters (Å^2^)

**Table:**

	*x*	*y*	*z*	*U*_iso_*/*U*_eq_	
Cl1	0.42007 (6)	0.86198 (3)	0.41239 (3)	0.02047 (11)	
O1	0.94601 (17)	0.35939 (10)	0.35994 (8)	0.01945 (19)	
N1	0.59391 (18)	0.43360 (11)	0.22863 (9)	0.0147 (2)	
C3	0.7047 (2)	0.59435 (13)	0.38427 (10)	0.0166 (2)	
H3A	0.8146	0.6130	0.4505	0.020*	
N3	0.2475 (2)	0.52018 (12)	0.09782 (10)	0.0179 (2)	
C1	0.3960 (2)	0.54346 (13)	0.19395 (10)	0.0140 (2)	
N2	0.33618 (18)	0.67556 (11)	0.24763 (9)	0.0140 (2)	
C2	0.4949 (2)	0.69401 (13)	0.34172 (10)	0.0144 (2)	
C4	0.7434 (2)	0.46134 (13)	0.32110 (10)	0.0151 (2)	
C5	0.9799 (3)	0.21688 (14)	0.30162 (12)	0.0227 (3)	
H5A	1.1372	0.1540	0.3359	0.034*	
H5B	1.0046	0.2451	0.2119	0.034*	
H5C	0.8208	0.1550	0.3175	0.034*	
O2	0.04847 (16)	0.09366 (10)	−0.17679 (8)	0.01766 (19)	
O3	0.18536 (16)	0.23812 (9)	−0.03536 (8)	0.01799 (19)	
C7	0.4170 (2)	−0.01172 (13)	−0.05581 (10)	0.0146 (2)	
H7A	0.5370	−0.0179	−0.1285	0.018*	
H7B	0.3287	−0.1133	−0.0381	0.018*	
C6	0.2070 (2)	0.12009 (13)	−0.08705 (10)	0.0135 (2)	
H1N3	0.286 (3)	0.439 (2)	0.0627 (15)	0.022 (4)*	
H2N3	0.123 (3)	0.588 (2)	0.0758 (15)	0.026 (4)*	
H1O2	−0.059 (3)	0.166 (2)	−0.1960 (16)	0.029 (4)*	

### Atomic displacement parameters (Å^2^)

**Table:**

	*U*^11^	*U*^22^	*U*^33^	*U*^12^	*U*^13^	*U*^23^
Cl1	0.02672 (17)	0.01570 (16)	0.02024 (16)	0.00612 (11)	−0.00563 (11)	−0.00917 (11)
O1	0.0221 (4)	0.0146 (4)	0.0220 (4)	0.0072 (3)	−0.0089 (3)	−0.0058 (3)
N1	0.0155 (4)	0.0127 (4)	0.0160 (5)	0.0016 (4)	−0.0023 (4)	−0.0032 (4)
C3	0.0196 (5)	0.0153 (5)	0.0153 (5)	0.0010 (4)	−0.0051 (4)	−0.0037 (4)
N3	0.0180 (5)	0.0156 (5)	0.0215 (5)	0.0057 (4)	−0.0071 (4)	−0.0094 (4)
C1	0.0131 (5)	0.0128 (5)	0.0162 (5)	−0.0004 (4)	0.0004 (4)	−0.0028 (4)
N2	0.0144 (4)	0.0127 (4)	0.0152 (4)	0.0018 (3)	−0.0014 (4)	−0.0037 (3)
C2	0.0179 (5)	0.0115 (5)	0.0143 (5)	0.0000 (4)	0.0008 (4)	−0.0035 (4)
C4	0.0154 (5)	0.0129 (5)	0.0163 (5)	0.0017 (4)	−0.0014 (4)	−0.0007 (4)
C5	0.0275 (6)	0.0142 (5)	0.0264 (6)	0.0086 (5)	−0.0071 (5)	−0.0064 (5)
O2	0.0176 (4)	0.0145 (4)	0.0214 (4)	0.0050 (3)	−0.0078 (3)	−0.0057 (3)
O3	0.0181 (4)	0.0146 (4)	0.0220 (4)	0.0040 (3)	−0.0055 (3)	−0.0064 (3)
C7	0.0139 (5)	0.0124 (5)	0.0178 (5)	0.0020 (4)	−0.0021 (4)	−0.0036 (4)
C6	0.0121 (5)	0.0131 (5)	0.0152 (5)	−0.0011 (4)	0.0007 (4)	−0.0013 (4)

### Geometric parameters (Å, º)

**Table:**

Cl1—C2	1.7370 (11)	N2—C2	1.3379 (15)
O1—C4	1.3379 (14)	C5—H5A	0.9800
O1—C5	1.4471 (14)	C5—H5B	0.9800
N1—C4	1.3184 (15)	C5—H5C	0.9800
N1—C1	1.3511 (14)	O2—C6	1.3191 (13)
C3—C2	1.3637 (16)	O2—H1O2	0.804 (19)
C3—C4	1.4075 (16)	O3—C6	1.2175 (14)
C3—H3A	0.9500	C7—C6	1.5080 (15)
N3—C1	1.3363 (15)	C7—C7^i^	1.525 (2)
N3—H1N3	0.846 (17)	C7—H7A	0.9900
N3—H2N3	0.842 (18)	C7—H7B	0.9900
C1—N2	1.3556 (14)		
			
C4—O1—C5	117.22 (9)	O1—C4—C3	116.16 (10)
C4—N1—C1	116.08 (9)	O1—C5—H5A	109.5
C2—C3—C4	113.88 (10)	O1—C5—H5B	109.5
C2—C3—H3A	123.1	H5A—C5—H5B	109.5
C4—C3—H3A	123.1	O1—C5—H5C	109.5
C1—N3—H1N3	117.9 (11)	H5A—C5—H5C	109.5
C1—N3—H2N3	117.7 (11)	H5B—C5—H5C	109.5
H1N3—N3—H2N3	124.4 (16)	C6—O2—H1O2	112.9 (12)
N3—C1—N1	117.06 (10)	C6—C7—C7^i^	112.44 (11)
N3—C1—N2	117.23 (10)	C6—C7—H7A	109.1
N1—C1—N2	125.71 (10)	C7^i^—C7—H7A	109.1
C2—N2—C1	114.50 (9)	C6—C7—H7B	109.1
N2—C2—C3	125.78 (10)	C7^i^—C7—H7B	109.1
N2—C2—Cl1	115.19 (8)	H7A—C7—H7B	107.8
C3—C2—Cl1	119.02 (9)	O3—C6—O2	123.52 (10)
N1—C4—O1	119.81 (10)	O3—C6—C7	123.89 (10)
N1—C4—C3	124.03 (10)	O2—C6—C7	112.59 (9)
			
C4—N1—C1—N3	177.94 (10)	C1—N1—C4—O1	−179.08 (9)
C4—N1—C1—N2	−1.63 (16)	C1—N1—C4—C3	0.99 (16)
N3—C1—N2—C2	−178.75 (10)	C5—O1—C4—N1	−3.63 (15)
N1—C1—N2—C2	0.81 (16)	C5—O1—C4—C3	176.31 (10)
C1—N2—C2—C3	0.73 (16)	C2—C3—C4—N1	0.32 (17)
C1—N2—C2—Cl1	−179.61 (7)	C2—C3—C4—O1	−179.62 (9)
C4—C3—C2—N2	−1.25 (17)	C7^i^—C7—C6—O3	5.35 (17)
C4—C3—C2—Cl1	179.10 (8)	C7^i^—C7—C6—O2	−174.64 (11)

Symmetry code: (i) −*x*+1, −*y*, −*z*.

### Hydrogen-bond geometry (Å, º)

**Table:**

*D*—H···*A*	*D*—H	H···*A*	*D*···*A*	*D*—H···*A*
N3—H1*N*3···O3	0.847 (17)	2.223 (17)	3.0055 (13)	153.7 (14)
N3—H2*N*3···O3^ii^	0.844 (16)	2.095 (16)	2.9369 (13)	175.4 (15)
O2—H1*O*2···N2^ii^	0.806 (16)	1.923 (16)	2.7266 (13)	174.6 (18)
C3—H3*A*···O1^iii^	0.95	2.45	3.3911 (14)	172

Symmetry codes: (ii) −*x*, −*y*+1, −*z*; (iii) −*x*+2, −*y*+1, −*z*+1.
